# The influence of multiple firing events on the formation and stability of activity patterns in continuous attractor networks

**DOI:** 10.1186/1471-2202-14-S1-P241

**Published:** 2013-07-08

**Authors:** David Lyttle, Alfredo Weitzenfeld, Jean-Marc Fellous, Kevin K Lin

**Affiliations:** 1 Program in Applied Mathematics, University of Arizona, Tucson, AZ 85721, USA; 2 Department of Mathematics, University of Arizona, Tucson, AZ 85721, USA; 3 Department of Psychology, University of Arizona, Tucson, AZ 85721, USA; 4 Department of Computer Science and Engineering, University of South Florida, Tampa, FL 33620, USA

## 

Continuous attractor networks have been proposed to explain a variety of phenomena, including working memory and rodent entorhinal grid cells [1,2]. Typically, such networks consist of spatially-structured lattices of neurons in one or two dimensions with long-range inhibition and short-range excitation, which causes the network activity to spontaneously self-organize into one or more "bumps" of persistent activity. In two-dimensions, the activity patterns often take the form of triangular lattices, which bear a striking resemblance to the spatial patterns observed in recordings of entorhinal grid cells [3]. Rate-based neural field models of attractor networks predict the emergence of stable, stationary patterns [2,4], whereas simulations of integrate and fire neurons suggest a more complicated dynamical picture. In particular, synaptic timescales appear to play a crucial role [5,6] in that fast synapses tend to lead to transient, local synchronous activity, thus destabilizing activity patterns. Recent work on a model of V1 suggests that such "multiple firing events" (MFEs) may be a generic, emergent feature of spiking networks [7]. In this work, we investigate whether and how MFEs may affect pattern stability in continuous attractor networks. While the stability of spiking neuron-based attractor networks have been studied previously [5,6], the models studied to date contain neither seperate excitatory and inhibitory populations nor a mixture of synaptic timescales (as would be expected in more realistic settings), and the effects of these features on pattern stability are not known.

To investigate these questions, we have implemented continuous attractor networks in both one and two dimensions using spiking, integrate-and-fire neurons with conductance-based synapses and seperate excitatory and inhibitory populations. Through simulations, we systematically assess the effect of MFEs on the formation and stability of spatiotemporal activity patterns ("bumps" and "grids"). We also investigate the effects of multiple synaptic timescales, noise, and network heterogeneity on the stability of these patterns. An overarching goal of this work is to obtain insights into how real neural systems might maintain stable persistent activity states, such as those needed for accurately integrating sensorimotor information. Furthermore, as 2-D attractor networks have been incorporated into a number of recent grid cell models [2,8], and the stability of grid cell activity may have a significant effect on place fields [9], understanding the stability of the grid patterns formed by these networks is highly relevant to studies of the rat spatial navigation system.
